# Effect of flowering stage and storage conditions on pollen quality of six male date palm genotypes

**DOI:** 10.1016/j.sjbs.2021.12.038

**Published:** 2021-12-17

**Authors:** Kadri Karim, Mohamed A. Awad, Abdelhafidh Manar, Jemni Monia, Aounallah Karim, Elsafy Mohammed

**Affiliations:** a Laboratory of biotechnology and oasis genetic resources, Regional Center of Research in the Agricultural Oasis of Degache, BP 62, km1 road of Tozeur, 2260 Degache, Tunisia; b National Institute of Agronomic Research of Tunis, Laboratory of Biotechnology applied on Agriculture, HediKarray street, 2049, Ariana, University of Carthage,Tunisia; c Department of Arid land Agriculture, Faculty of Meteorology, Environment and Arid land Agriculture, King Abdulaziz University, P.O.Box. 80208, Jeddah, Saudi Arabia; d Pomology Department, Faculty of Agriculture, Mansoura University, El-Mansoura, Egypt; e National Institute of Agricultural Sciences of Tunis, 43, Charles NICOLE street 1082, City of Mahrajen, Tunis, Tunisia; f Department of Plant Breeding, Swedish University of Agricultural Sciences. Alnarp, Skåne, Sweden

**Keywords:** *Phoenix dactylifera* L., Pollination, Male inflorescence, Pollen quality, Pollen storage

## Abstract

Availability of efficient male genotypes is critical for successful artificial pollination and regular bearing of female date palms. The effect of flowering stage and storage conditions on pollen quality of six male date palm genotypes encoded ‘ABD1′, ‘P4′, ‘P3′, ‘P8′, ‘P7′ and ‘P13′were evaluated. Pollen collected from spathes developed at the middle of flowering stage exhibited the best viability (90%) and germinability (85%) compared to other stages. Pollen viability was greater than 90%, except for ‘P8′ that exhibited 80%, while, germinability greatly varied among the genotypes. Pollen quality decreased during 4 months of storage upon genotype and temperature, with a minimum reduction at −30 °C followed by 4 °C. Heat shock exposure (33 ± 2 °C) following storage revealed that pollen stored at −30 °C or 4 °C should be used for pollination on the same day of take out to avoid dramatic quality loss. The ‘ABD1′, an early flowering genotype, proved highest pollen quality both at fresh stage and after storage. While, the ‘P3′, a late flowering genotype, retained its pollen quality during storage. However, the ‘P13′ genotype exhibited excellent pollen quality when fresh, but greatly loses germinability during storage.

## 1 Introduction

Availability of efficient male pollinators are of great importance in date palm (*Phoenix dactylifera* L.) production chain and for regular yearly bearing as the quantity and quality of pollen is a yield-determining factor (Mesnoua et al., 2018, Kadri et al., 2019). The flowering and pollination period of date palm varies upon cultivar, geographic location and climatic conditions. However, frequent asynchronous flowering of date palm male and female trees occur due to climatic changes and abiotic stress (Mesnoua et al., 2018, Kadri et al., 2019). In such cases, farmers may pollinate their trees with pollen of a known male conserved at ambient conditions from the previous season; however, this is mostly result in a low fruit set and yield (Rezazadeh et al., 2013). Alternatively, they may use available pollens from un-tested male genotypes that may cause low fruit set and/or have negative metaxenic effects on fruit quality (Maryam et al., 2015, Kadri et al., 2019). Generally, date palm male trees produce their spathes (inflorescences) in stages or waves during the flowering period that mostly extend to more than a month. However, there is little available information on pollen quality from spathes developed at the different times (Bounaga, 1991). In this respect, it has been reported that pollen of six Algerian male date palm cultivars lost most of their viability after 2–3 months of storage at room temperature, in contrast to those stored at −20 °C, with great variation among genotypes (Mesnoua et al., 2018). Pollen grains conditioning following storage is also a critical aspect, since heat shock exposure ultimately reduce pollen quality due to the dehydration and ultra-structural modifications of the pollen envelope (Buitink et al., 2000, Mizrahi et al., 2000, Akond et al., 2012). The current study aim to (1) evaluate pollen quality of inflorescences developed at three different flowering stages, (2) evaluate the effect of storage duration at different temperatures on pollen quality, and (3) estimate the impact of heat shock exposure (33 ± 2 °C) on pollen quality following storage for six male date palm genotypes.

## 2 Materials and methods

### 2.1 Plant materials

**Fig. 1:**
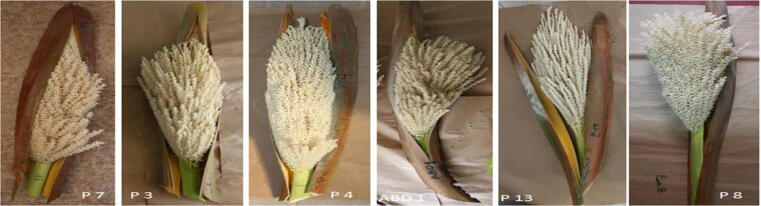
Spathe image of the six male date palm genotypes used in this study.

This experiment was conducted on six male date palm genotypes growing at the experimental plot of the Regional Center of Research in The Agricultural Oasis of Degache located at the South West of Tunisia. These genotypes encoded ‘ABD1′, ‘P4′, ‘P3′, ‘P8′, ‘P7′ and ‘P13′ (Fig. 1) and received the regular agricultural practices used at the region. The characteristics of these genotypes including flowering time, total number of spathe produced and percentage of spathe production at each flowering stage were described (Table 1). From each genotype, three samples of fresh pollen were separately and randomly collected from different inflorescences at different stages (early, medium and late, as the beginning of spathe cracking) for viability and germinability tests, conductivity and pH measurements as described below.

**Table 1:** Flowering time, total number of spathe produced and percentage of spathe production at each flowering stage of six date palm genotypes.

Male genotype		Floweringtime	Number of spathe produced	Spathe produced at each flowering stage (%)		
				Stage 1	Stage 2	Stage 3
ABD1		Early	33	33.33	30.30	36.36
P4		Early	36	33.33	33.33	33.33
P7		Early	36	27.77	38.88	33.33
P8		Early	33	33.33	27.27	39.39
P13		Medium	37	32.43	37.83	29.72
P3		Late	30	26.66	40.00	33.33

### 2.2 Pollen viability test

Pollen viability evaluation was conducted in triplicate samples by staining the fresh and stored pollens in slide with 1–2 drops of 1% acetocarmine dye with a coverslip and viewing them under a microscope with a magnification of 400X using the FuturJoe software as previously described (Kadri et al., 2017). The normal appearance of red-stained pollen grains were considered viable while the colorless wrinkled ones were recorded as non-viable, using 5 microscopic fields per slide and three replications for each source of pollen. Viability (%) was calculated using the formula: Viability (%) = (number of viable pollen grain/total number of grain) × 100.

### 2.3 Pollen germinability test

Evaluation of pollen germination was performed in triplicate samples using a standard media consisting of (Ca (NO_3_)2·.2H_2_O (0.20 g), H3BO_3_ (0.20 g), KNO_3_ (0.10 g), MgSO_4_·7H_2_O (0.20 g), agar (10 g) and sugar (150 g) per liter (Brewbaker and Kwack, 1964, Asif et al., 1983). The pH of the media was adjusted to 5.7 before agar addition and then sterilized in the autoclave for 20 min. Following that, the media was poured into Petri dishes under a laminar air flow hood and placed at room temperature to settle and cool. The fresh and stored pollens were sown on the dishes using brushes to have well separated pollens and incubated at 28 °C for 24 h. The germination of the fresh and stored pollens was evaluated using a microscope (400X magnification) and an average of five microscopic fields of vision was achieved for each storage temperature (Boughediri et al., 1995). The pollen germination percentage was calculated as follows: Germination (%) = (germinated pollen/total number of grains) × 100.

### 2.4 Electrical conductivity (EC) measurement of pollen filtrate

A mixture of fresh pollen (30 mg) and deionized water (10 ml) was prepared and incubated for 16 h in an oven at 28 °C (Ching & Ching, 1976). EC of pollen filtrate was measured using a Consort C6010 + conductivity meter that was previously calibrated.

### 2.5 pH measurement of pollen filtrate

The used technique involves mixing 250 mg of fresh pollen with 3 ml of distilled water in a 15 ml tube and the mixture was well vortexed, centrifuged for 10 min and then the pH was measured in the supernatant using a previously calibrated Consort C6010 + pH meter (Halimi, 2004).

### 2.6 Pollen collection and storage at different temperatures

Spikelets containing the fresh pollen grains were dried on kraft paper in a place protected from sunlight and wind. Pollens were extracted from these dried spikelets by manual shaking over Kraft paper, and then passed through a sieve to separate out the waste of other flower parts. The amount of pollen collected from each male genotype was divided over three hermetically sealed glass boxes. One box was stored in the refrigerator at 4 °C, the second in the freezer at − 30 °C and the third one was kept in an incubator at 28 °C. To avoid the effect of heat shock, the boxes placed at low temperatures (4 and − 30 °C) were aliquoted into small glass boxes each containing 2 to 3 g of pollens sufficient for each further individual periodical measurements.

### 2.7 Effect of storage duration at different temperature on pollen quality

The effects of storage duration (60, 75, 90, 105, and 120 days) at different temperature (28, 4 and − 30 °C) on pollen viability and germinability of the six male date palm genotypes were studied in vitro using the protocols described above.

### 2.8 Evaluation of heat shock exposure following storage on pollen quality

The effects of heat shock exposure (33 ± 2 °C) for different periods following four months of storage at different temperatures (28, 4 and − 30 °C) on pollen viability and germinability of the six male date palm genotypes were in vitro evaluated. The protocol consists of exposing the boxes of pollen grain to room temperature, and then viability and germinability tests were conducted every 48 h and during 20 days period using the protocols described above.

### 2.9 Statistical analyses

The data were statistically analyzed as a completely randomized design (CRD) with three replicates by analysis of variance (ANOVA) using the statistical package software ‘STATISTICA’ version 5. Comparisons between means were made by the least significant differences (LSD) at *P ≤* 5%.

## 3 Results

### 3.1 Physiological characterization of pollen from different genotypes and flowering stages

#### 3.1.1 Viability percentage

**Fig. 2:**
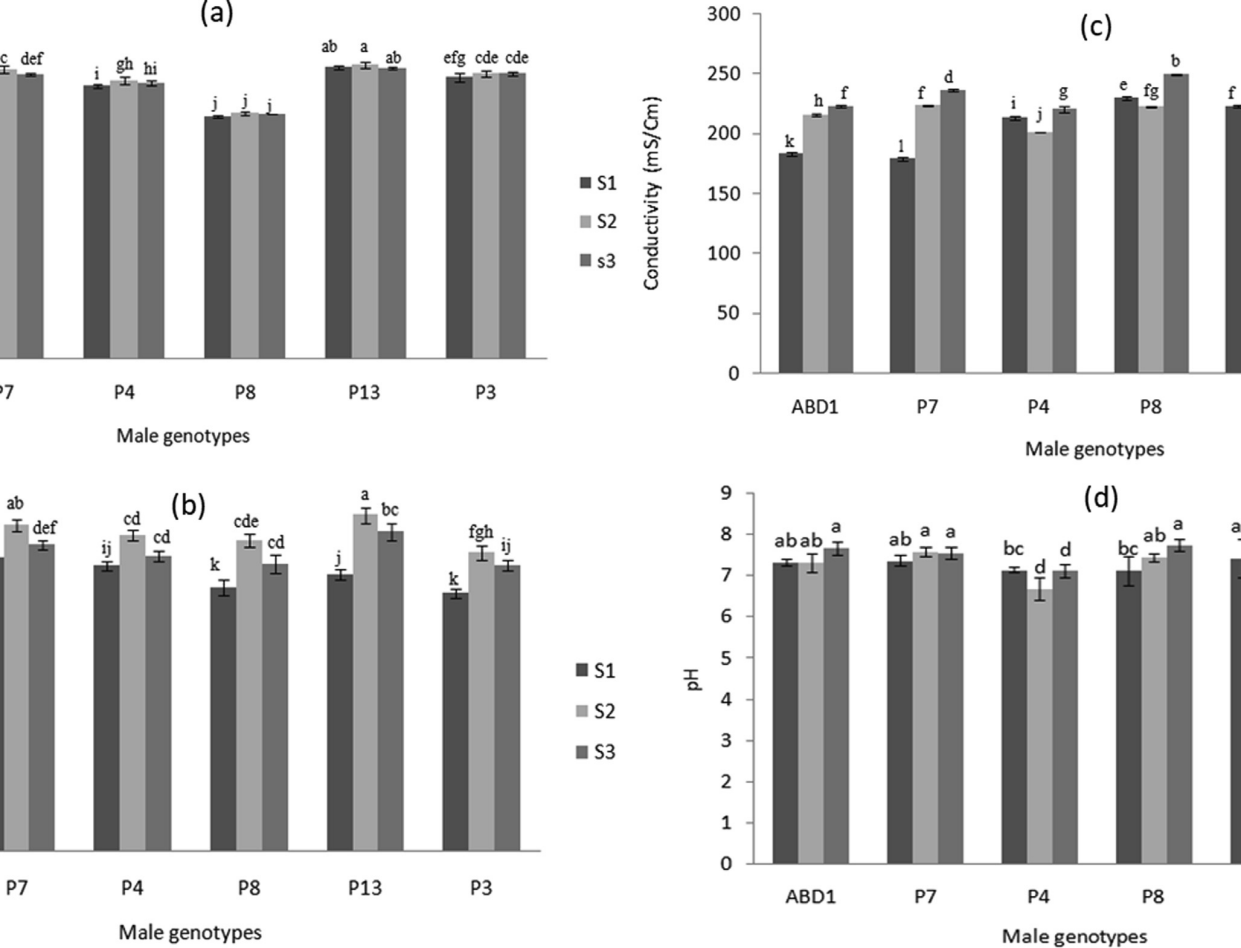
Variation in (a) pollen viability, (b) pollen germinability, (c) pollen conductivity, and (d) pH among six male date palm genotypes collected at different flowering stages. (S1), early-stage; (S2), middle stage; and (S3), late stage. Values are means ± SD (n = 3). Statistical analysis was performed using the LSD test at P ≤ 5%.

Almost all of the six male genotypes showed pollen viability greater than or equal to 90%, except for the male ‘P8′ that exhibited 80% (Fig. 2a). There were no differences in pollen viability among the various flowering stages. However, there were slight, but significant, differences in pollen viability among various genotypes. The highest viability was recorded in ‘P13′ (96.3%) followed by ‘P7′ (94.8%) and the lowest was in ‘P8′ (80.5%) genotype (Fig. 2a).

#### 3.1.2 Germinability percentage

Significant variations in pollen germinability were recorded among the genotypes and the flowering stages (Fig. 2b). Germination varied from 65% (‘P3′ S1) to 89.1% (‘P13′ S2). The highest germinabilities were found in the genotypes ‘P13′ (89.1%) and ‘P7′ (86.5%), while the lowest detected in ‘P3′ (65%). In all genotypes, spathes that were ripened at the middle of the flowering period exhibited the highest germinability compared to early and late stages (Fig. 2b).

#### 3.1.3 EC of pollen filtrate

The lowest EC of pollen filtrate obtained from the early flowering stage and ranged from 178.88 ms/cm in ‘P7′ up to 229.62 ms/cm in ‘P8′ genotype (Fig. 2c). The ‘P8′ genotype showed the lowest viability and germinability than other genotypes.

#### 3.1.4 pH of pollen filtrate

pH of pollen filtrate ranged from 6 to 7.5 and was significantly affected by genotype and flowering stage (Fig. 2d). The highest pH (7.68) obtained from the genotype ‘ABD1′ while the lowest (6.67) obtained from ‘P3′. The genotypes ‘P3′ (at all flowering stages) and ‘P4′ (at medium and late flowering stages) showed lower pH values than those of ‘ABD1′, ‘P7′, ‘P8′ (at medium and late flowering stages) and ‘P13′ (at early flowering stage).

### 3.2 Effect of storage duration at different temperature on pollen quality

#### 3.2.1 Evaluation of pollen viability

**Fig. 3:**
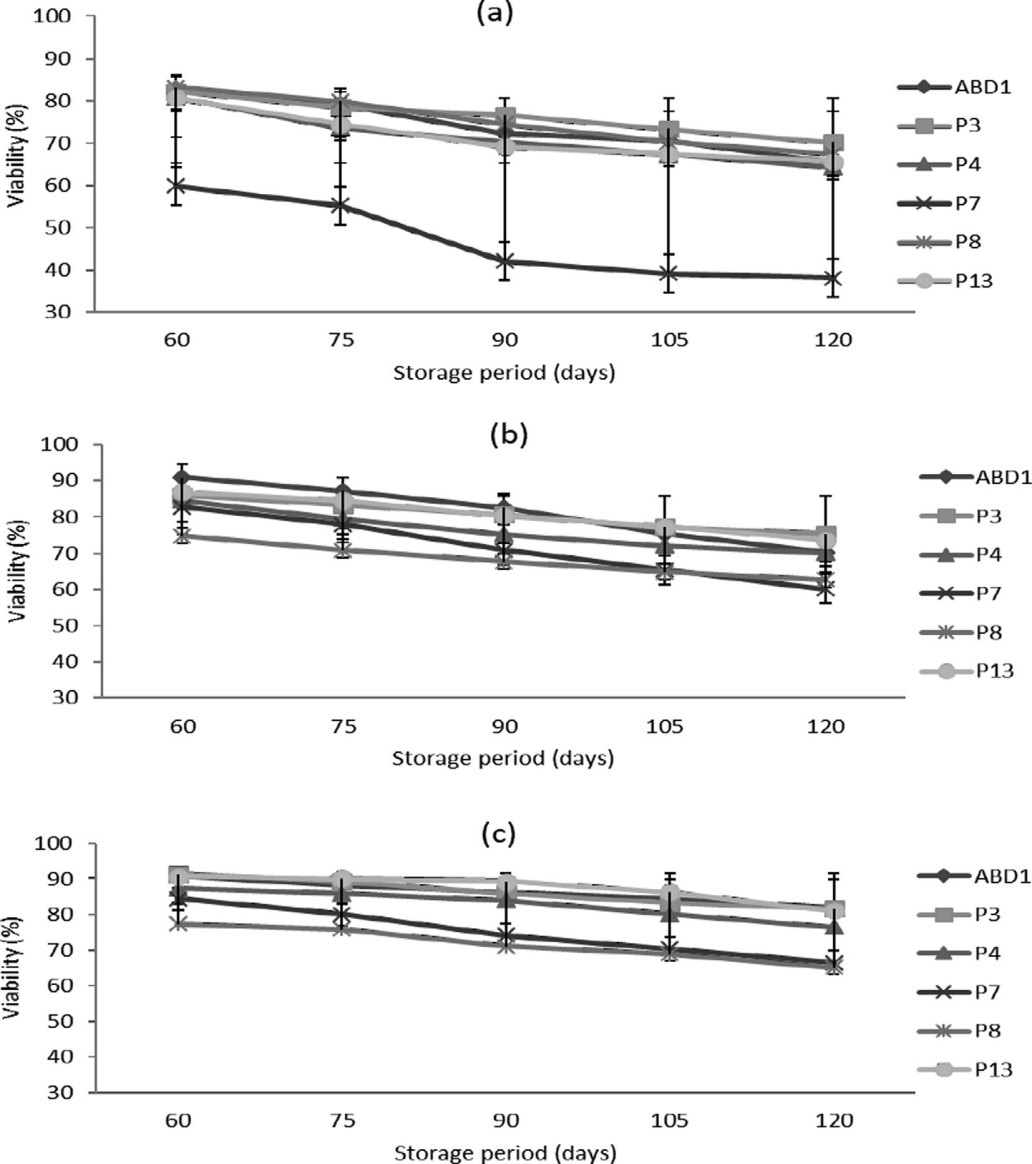
Effect of storage period at different temperatures on pollen viability of six male date palm genotypes. (a), pollen stored at 28 °C; (b), pollen stored at 4 °C; and (c), pollen stored at −30 °C. Values are means ± SD (n = 3). LSD test at P ≤ 5% for storage period and genotype interaction = 0.66, 0.62 and 3.59 at 28, 4 and −30 °C, respectively.

Compared to the initial levels, the rate of reduction in viability of pollens stored at 28 °C varies between 5 and 15%, but it did not exceed 6% for those stored at 4 °C after 60 days of storage (Fig. 3; Table 2). However, pollen stored at − 30 °C retained their viability almost constant. The genotype ‘ABD1′ exhibited the highest viability (93.5%) followed by ‘P7′ (92.7%), however the lowest viability was detected in ‘P8′ (79.2%). After 120 days of storage, a significant decrease in the viability was recorded especially for the pollen stored at 28 °C, with a reduction average of up to 35% or even 60% as observed in the ‘P7′ genotype. However, there were only slight change in pollen viability stored at 4 °C and the majority of genotypes retained an average viability around 70% with the exception of ‘P8′, which showed a reduction of 33%. On the hand, pollen stored at − 30 °C, for 4 months retained their higher viability with a reduction rate of<15%, except for the genotype ‘P8′ that showed a higher reduction (31.1%).

**Table 2:** Mixed factorial design showing the effect of storage duration (x5) and genotype (x6) at different temperatures and on in vitro pollen viability and germinability of six date palm genotypes.

Viability (Fig. 3 a, b, c)									
Source	F-value			Anova P-value			LSD value		
	28 °C	4 °C	−30 °C	28 °C	4 °C	−30 °C	28 °C	4 °C	−30 °C
Storage duration	4892.1	6544.1	119.6	<0.0000	<0.0000	<0.0000	0.240	0.226	1.309
Genotype	24537.9	4506.1	182.0	<0.0000	<0.0000	<0.0000	0.267	0.251	1.452
Storage duration × Genotype	113.4	133.5	2.46	<0.0000	<0.0000	<0.0000	0.660	0.621	3.590
Germinability (Fig. 4 a, b, c)									

Source	F-value			Anova P-value			LSD value		
	28 °C	4 °C	−30 °C	28 °C	4 °C	−30 °C	28 °C	4 °C	−30 °C

Storage duration	7261.9	1848.5	5732.0	<0.0000	<0.0000	<0.0000	0.230	0.492	0.223
Genotype	17202.9	605.3	2217.5	<0.0000	<0.0000	<0.0000	0.255	0.546	0.247
Storage duration × Genotype	115.5	25.4	35.8	<0.0000	<0.0000	<0.0000	0.632	1.349	0.612

#### 3.2.2 Evolution of pollen germinability

**Fig. 4:**
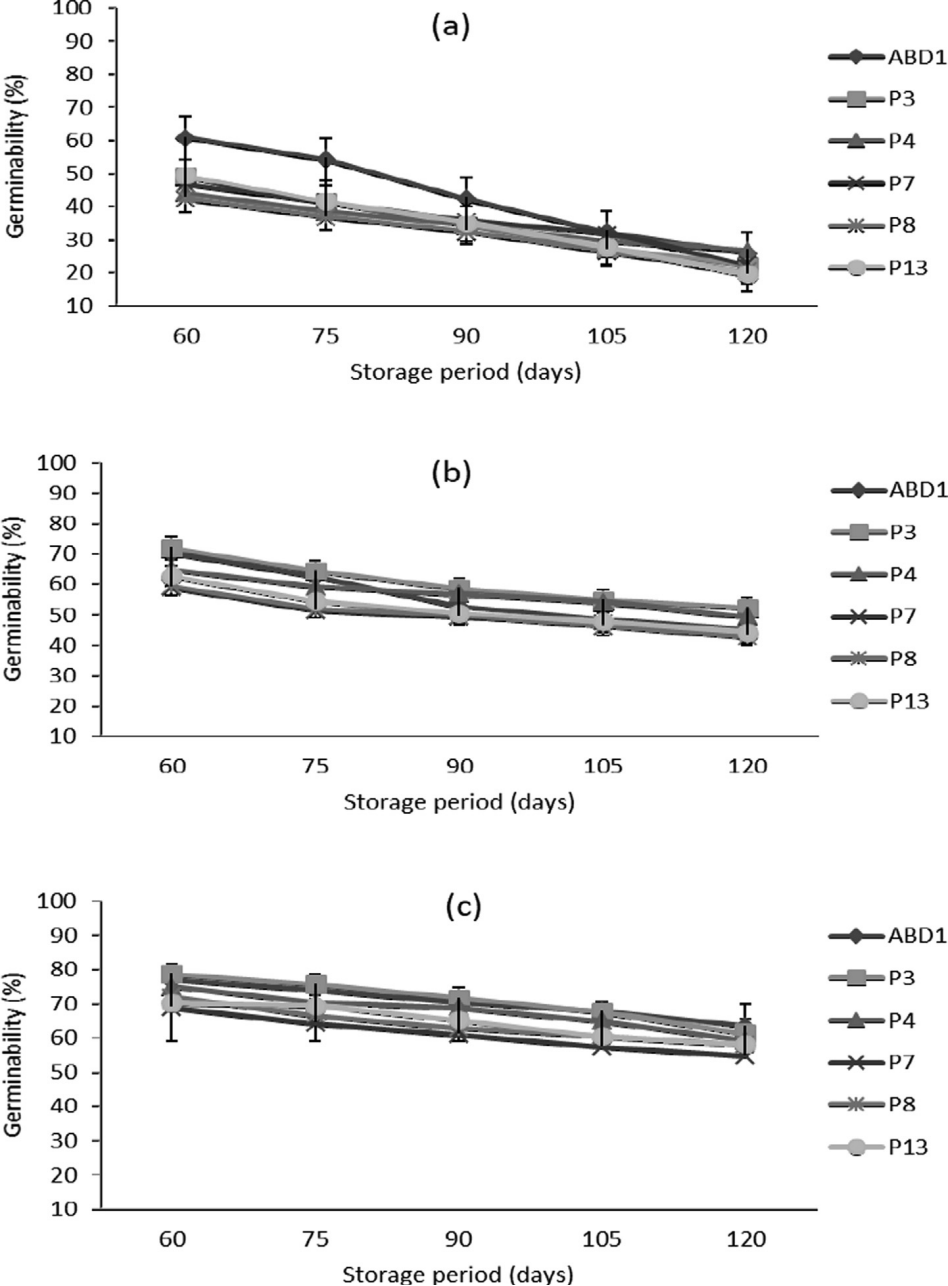
Effect of storage period at different temperatures on pollen germinability of six male date palm genotypes. (a), pollen stored at 28 °C; (b), pollen stored at 4 °C; and (c), pollen stored at −30 °C. Values are means ± SD (n = 3). LSD test at P ≤ 5% for storage period and genotype interaction = 0.63, 1.34 and 0.61 at 28, 4 and −30 °C, respectively.

Pollen stored at room temperature recorded reductions in germinability varying between 24 and 46% for the six male genotypes (Fig. 4; Table 2). After 60 days of storage at 4 °C, the highest germinabilitiy was registered in the genotype ‘P3′ (71%) followed by ‘ABD1′ (70%) while the lowest detected in the genotypes ‘P7′ (58 %) and ‘P8′ (59%). However, pollen stored at − 30 °C retained higher germinabilitiy that vary between 68% in ‘P7′ and 78% in ‘P3′ genotypes with a fairly low reduction level averaged 10% in ‘ABD1′ and 20% in ‘P13′ genotypes compared to fresh pollen. After 90 days of storage, pollen stored at 28 °C recorded a reduction level up to 68.75% with the genotype ‘P13′, indicating a remarkable degradation of pollen quality. However, for pollen stored at 4 °C, the decrease in germinabilitiy was between 34% and 50%. While, such decrease was much less pronounced in pollen stored at − 30 °C especially for the genotypes ‘ABD1′ and ‘P3′ that showed a reduction of only 21% in contrast to the genotype ‘P13′ that 31%.

### 3.3 Effect of heat shock following storage on pollen quality

#### 3.3.1 Effect of heat shock on pollen viability

**Fig 5:**
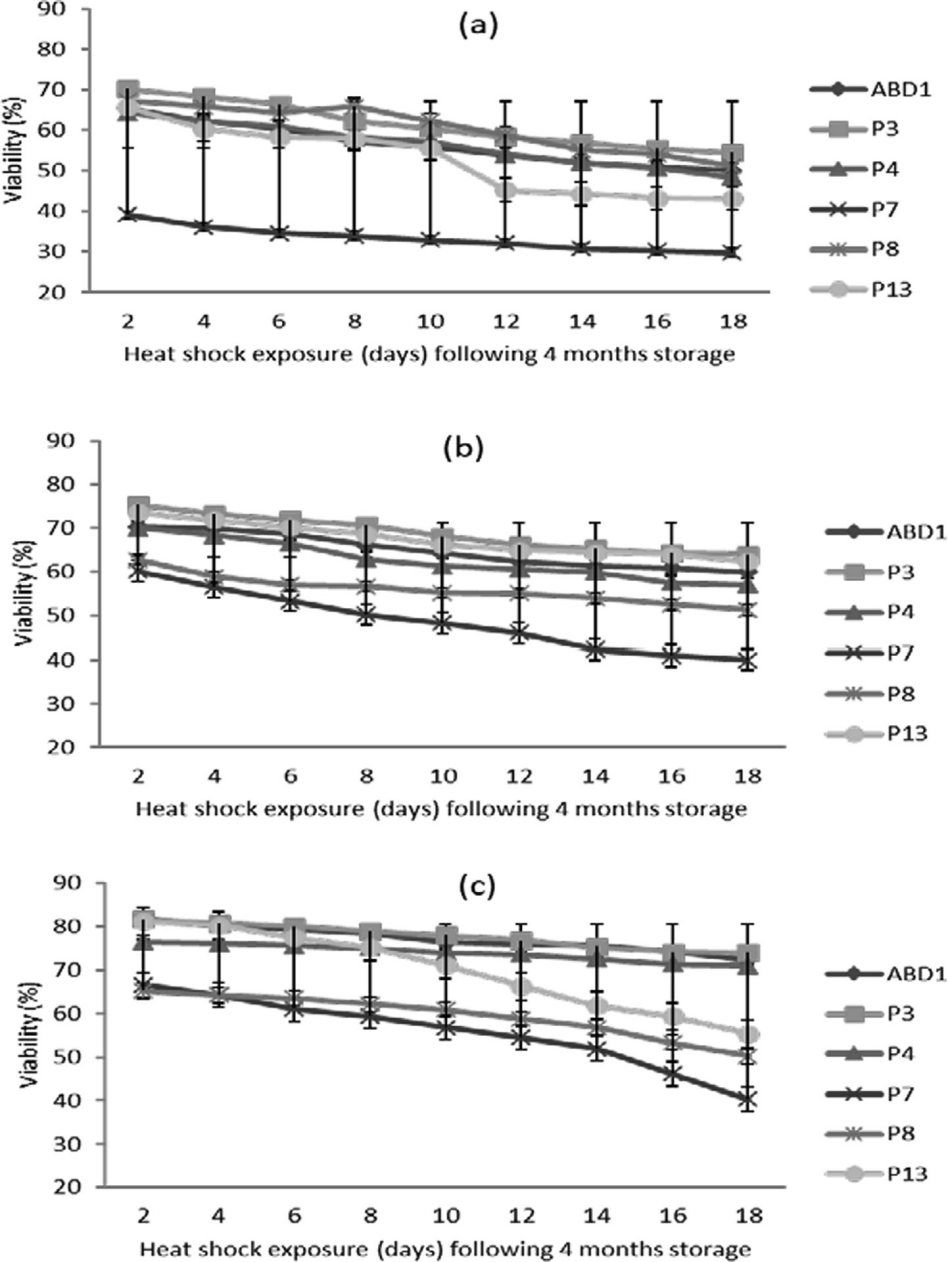
Effect of heat shock (33 ± 2 °C) exposure for different durations following 4 months of storage at different temperatures on pollen viability of six male date palm genotypes. (a), pollen stored at 28 °C; (b), pollen stored at 4 °C; and (c), pollen stored at −30 °C. Values are means ± SD (n = 3). LSD test at P ≤ 5% for heat shock period and genotype interaction = 0.68, 0.63 and 1.02 at 28, 4 and −30 °C, respectively.

A gradual reduction in pollen viability was observed throughout 20 days of exposure to heat shock (33 ± 2 °C) following storage for each genotype. For the first 48 h, a viability loss ranged between 1.5% and 5% was reordered (Fig. 5; Table 3). It tends to increase with the longer exposure duration to heat reaching a maximum loss of around 22.5% for ‘P13′ and a minimum level of about 9.3% with ‘P7′ after 20 days following storage at 28 °C (Fig. 5a). While, such viability loss during 20 days of heat shock exposure following storage at 4 °C ranged between 10 and 20% with the lowest level in ‘ABD1′ (10.4%) and the highest in ‘P7′ (20.2%) genotype (Fig. 5b). However, viability loss was more remarkable for pollen that were stored at − 30 °C reaching about 26% after 20 days of heat shock exposure (Fig. 5c).

**Table 3:** Mixed factorial design showing the effect of heat shock exposure duration (x9) and male date palm genotype (x6) following storage at different temperatures on in vitro pollen viability and germinability.

Viability (Fig. 5 a, b, c)									
Source	F-value			Anova P-value			LSD value		
	28 °C	4 °C	−30 °C	28 °C	4 °C	−30 °C	28 °C	4 °C	−30 °C
Heat shock	4892.1	3681.8	1796.9	<0.0000	<0.0000	<0.0000	0.251	0.231	0.377
Genotype	24537.9	16039.4	8651.1	<0.0000	<0.0000	<0.0000	0.199	0.183	0.299
Heat shock × Genotype	113.4	69.9	127.6	<0.0000	<0.0000	<0.0000	0.683	0.630	1.026
Germinability (Fig. 6 a, b, c)									

Source	F-value			Anova P-value			LSD value		
	28 °C	4 °C	−30 °C	28 °C	4 °C	−30 °C	28 °C	4 °C	−30 °C

Heat shock	2944.3	12591.1	45425.2	<0.0000	<0.0000	<0.0000	0.259	0.252	0.214
Genotype	1148.6	1348.8	2410.8	<0.0000	<0.0000	<0.0000	0.206	0.200	0.170
Heat shock × Genotype	34.6	94.3	185.4	<0.0000	<0.0000	<0.0000	0.706	0.686	0.583

#### 3.3.2 Effect of heat shock following storage on pollen germinability

**Fig. 6:**
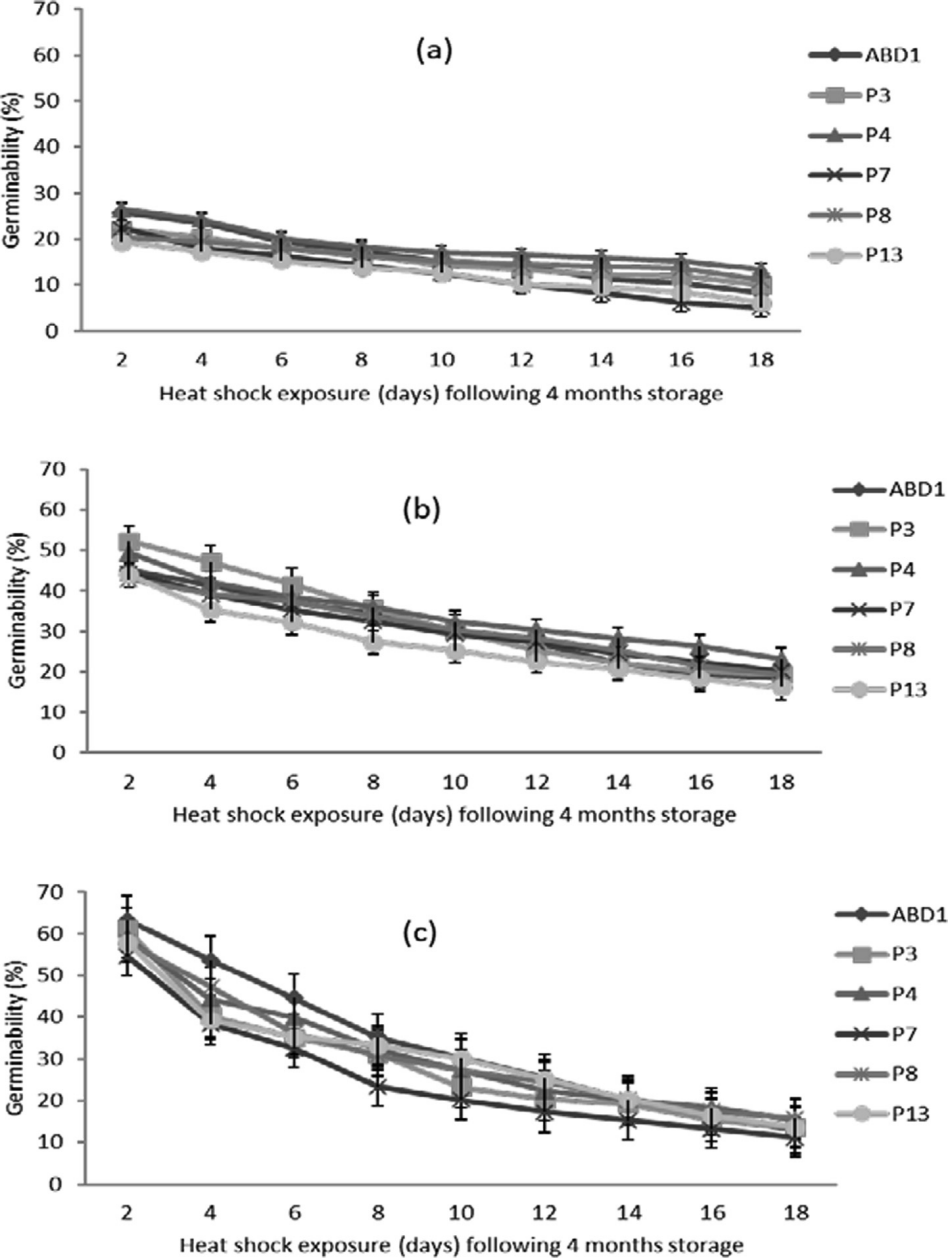
Effect of heat shock (33 ± 2 °C) exposure for different durations following 4 months of storage at different temperatures on pollen germinability of six male date palm genotypes. (a), pollen stored at 28 °C; (b), pollen stored at 4 °C; and (c), pollen stored at −30 °C. Values are means ± SD (n = 3). LSD test at P ≤ 5% for heat shock period and genotype interaction = 0.70, 0.68 and 0.58 at 28, 4 and −30 °C, respectively.

After 20 days, pollen stored at 28 °C, recorded a small germinability reduction ranged between 9.3% for ‘P8′ and 17.6% for ‘ABD1′ genotypes (Fig. 6a; Table 3). These results show that the heat shock does not have a great effect on germinability of pollen stored at ambient temperature. However, pollen stored in the refrigeration (4 °C), undergoes a dramatic decrease in germinability during the 20 days of heat shock (Fig. 6b). The greatest reduction in germinability (63.9%) was recorded in the genotype ‘P3′ while, the lowest (53.4%) was detected in ‘P4′ with an average of 50% for the other genotypes. However, the reduction in terms of germinability was very critical for the pollen stored at − 30 °C (Fig. 6c). The genotype ‘P3′ recorded 33% reduction during only the first 2 days of heat shock. Two weeks later, the reduction in germinability reached a maximum level (79.3%) for the genotype ‘ABD1′ and a minimum level (74.4%) for the ‘P4′ genotype, with an average reduction of 77% for the other genotypes.

## 4 Discussion

### 4.1 Physiological characterization of pollen from different genotypes and flowering stages

The in vitro evaluation of pollen viability revealed significant differences among various genotypes and among various flowering stages. The genotypes ‘P13′ and ‘P7′ exhibited the highest viability and germinability. However, the lowest viability was recorded in ‘P8′ but the lowest germinability was detected in ‘P3′ genotype. These results partly confirm previous studies on other date palm genotypes regarding genotype variations (Shaheen et al., 1986, Kadri et al., 2017). Interestingly, the genotype ‘P3′ showed a high viability (92.3%) however, exhibited much lower germinability (69%), confirming no positive correlation between pollen viability and germinability percentage for date palm as previously reported for *E. Japonica* (Yang et al., 2015). The germinability of date palm pollen is considered a critical indicator to guarantee a commercial fruit set and yield (Mesnoua et al., 2018, El Kadri and Ben Mimoun, 2020). The germination percentages between 60 and 75% of pollen in *in vitro* test were required to ensure commercial fruit set (Peyron, 2000, Mesnoua et al., 2018). Interestingly, in all genotypes, spathes that were ripened at the middle of the flowering period exhibited the highest germinability compared to early and late stages. Our results confirm previous study in which both early and late developed spathes give pollen of poor quality compared to those developed in the middle of the flowering period (Munier, 1973). The results of EC of pollen filtrate revealed that the ‘P8′ genotype showed the lowest viability and relatively lower germinability compared with the other genotypes. It is considered that higher EC reflects a massive electrolyte leakage and attribute to poor pollen integrity membrane (Colas and Mercier, 2000). Our results are in agreement with previous study on other male date palm genotypes (Babahani and Bouguedoura, 2009, Kadri et al., 2017). The pH measurement of pollen filtrate ranged from 6 to 7.5 with significant variations among genotypes and flowering stages. However, previous studies showed that the pH of fresh pollen was mostly close to neutral and that was not a distinguishing criterion of pollen quality among genotypes (Babahani and Bouguedoura, 2009, Kadri et al., 2017, El Kadri and Ben Mimoun, 2020).

### 4.2 Effect of storage duration at different temperature on pollen quality

The results of the current study revealed that storage temperature and duration are crucial for date palm pollen viability and germinability. In this respect, pollen quality gradually decreased during 4 months of storage upon genotype and temperature, with a minimum reduction at − 30 °C followed by 4 °C. Pollen of the ‘ABD1′ genotype proved highest quality both at fresh stage and after storage. However, it is considered a precocious compared to female cultivars such as ‘Deglet Nour’ that commonly growing in the region, indicating a need for pollen storage. The genotype ‘P3′ genotype preserved its pollen quality during storage better than most of the other genotypes but flowering somewhat later than the female ‘Deglet Nour’ cultivar. However, the ‘P13′ genotype exhibited excellent pollens quality when fresh, but loses their quality during storage. It has been reported that storage of pollen at room temperature (28 °C) greatly reduced pollen viability and germinability as well as fruit set and yield of date palm (Kadri et al., 2017). In addition, the variation in pollen storability among the different genotypes are consistent with previous studies (Halimi, 2004, Ateyyeh, 2012, Rezazadeh et al., 2013). In another study, pollen stored at room temperature (24 °C ± 2) lost more than 95% of their quality after 2 to 3 months in various genotypes after which pollen quality was totally lost with longer storage duration (Mesnoua et al., 2018). Recently, similar results were also reported in which the maximum germinability was obtained with storage at − 20 °C followed by 4 °C for several Tunisian (El Kadri and Ben Mimoun, 2020) and Algerian (Mesnoua et al., 2018) male date palm genotypes. In addition, our results confirm previous studies where the optimum pollen storage temperature varies between − 20 °C to −80 °C, which limit the ongoing metabolic activities in pollen (Mortazavi et al., 2010, Maryam et al., 2015, Kadri et al., 2017). Moreover, cryogenic storage (−196 °C) was suggested as a promising method to store date palm pollen for commercial date production, breeding programs and conservation of elite pollen genotypes (Anushma et al., 2018). The variation in pollen germinability among genotypes during storage has been attributed to their tolerance to desiccation, which might be genetically determined (Chao and Krueger, 2007, Akond et al., 2012, Mesnoua et al., 2018).

### 4.3 Effect of heat shock following storage on pollen quality

Our results revealed that the heat shock (33 ± 2 °C) exposure following storage does not have a great effect on quality of pollen stored at ambient temperature, in contrast to those stored at − 30 °C or 4 °C, that undergoes a dramatic decrease during 20 days of heat shock. Accordingly, pollen stored at − 30 °C or 4 °C should be used for pollination on the same day of take out to avoid rapid quality loss. These results are possibly due to the large difference between the previous storage temperature and heat shock that led to rapid dehydration of pollen (Mizrahi, 2000). To minimize the effects of heat shock, the rehydration process following storage is critical, since this process allow the plasmalemma to regain its normal structure and ensure a normal recovery of metabolic activities (Buitink et al., 2000). Such quality losses might be attributed to the dehydration and ultra-structural modifications of the pollen envelope following cold or freezing storage (Mesnoua et al., 2018, Akond et al., 2012).

## 5 Conclusion

The results of this study revealed that pollen of the six date palm male genotypes collected at the middle flowering stage exhibited the highest viability and germinability compared to those collected at early or late stages. Storing date palm pollen at − 30 °C retained pollen viability and germinability much better than at 4 °C or 28 °C. Pollen stored at − 30 °C or 4 °C should be used for pollination on the same day of take out to minimize quality loss due to heat shock exposure. Pollen of the ‘ABD1′ genotype proved highest quality both at fresh stage and after storage. However, it is considered a precocious compared to female cultivars such as ‘Deglet Nour’ that commonly growing in the region, indicating the need of pollen storage. The genotype ‘P3′ preserved its pollen quality during storage better than most of the other genotypes but flowering somewhat later than the female ‘Deglet Nour’ cultivar. However, the ‘P13′ genotype exhibited excellent pollen quality when fresh, but loses their quality during storage.

## Declaration of Competing Interest

The authors declare that they have no known competing financial interests or personal relationships that could have appeared to influence the work reported in this paper.
